# Exploring the Determinants of the Severity of Pedestrian Injuries by Pedestrian Age: A Case Study of Daegu Metropolitan City, South Korea

**DOI:** 10.3390/ijerph17072358

**Published:** 2020-03-31

**Authors:** Seung-Hoon Park, Min-Kyung Bae

**Affiliations:** 1 Department of Urban Planning, Keimyung University, Daegu 42601, Korea; 2 Land & Housing Institute, Daejeon 34047, Korea; bimin0224@naver.com

**Keywords:** pedestrian-vehicle crash, severity of pedestrian injury, built environment, pedestrian safety

## Abstract

Pedestrian-vehicle crashes can result in serious injury to pedestrians, who are exposed to danger when in close proximity to moving vehicles. Furthermore, these injuries can be considerably serious and even lead to death in a manner that varies depending on the pedestrian’s age. This is because the pedestrian’s physical characteristics and behaviors, particularly in relation to roads with moving vehicles, differ depending on the pedestrian’s age. This study examines the determinants of pedestrian injury severity by pedestrian age using binary logistic regression. Factors in the built environment, such as road characteristics and land use of the places where pedestrian crashes occurred, were considered, as were the accident characteristics of the pedestrians and drivers. The analysis determined that the accident characteristics of drivers and pedestrians are more influential in pedestrian-vehicle crashes than the factors of the built environmental characteristics. However, there are substantial differences in injury severity relative to the pedestrian’s age. Young pedestrians (aged under 20 years old) are more likely to suffer serious injury in school zones; however, no association between silver zones and injury severity is found for elderly pedestrians. For people in the age range of 20–39 years old, the severity of pedestrian injuries is lower in areas with more crosswalks and speed cameras. People in the age range of 40–64 years old are more likely to be injured in areas with more neighborhood streets and industrial land use. Elderly pedestrians are likely to suffer fatal injuries in areas with more traffic signals. This study finds that there are differences in the factors of pedestrian injury severity according to the age of pedestrians. Therefore, it is suggested that concrete and efficient policies related to pedestrian age are required to improve pedestrian safety and reduce pedestrian-vehicle crashes.

## 1. Introduction

Walking is the most basic means of transportation [1,2]. Its importance for improving public health, saving energy, and reducing traffic congestion and air pollution has long been acknowledged. However, because of a steady increase in the number of pedestrian-vehicle crashes, the safety of walking is not guaranteed [3]. In 2016, approximately 1.4 million people around the world died in pedestrian-vehicle crashes [4].

South Korea featured the second-highest pedestrian death rate among the OECD countries, with 10.8 road fatalities per 100,000 population in 2012 [5]. In response, the country has acted on a national scale to reduce both this figure and this type of accident overall. As a result, the number of pedestrian-vehicle crashes has decreased gradually, beginning in 2015; in 2017, the country recorded the lowest number of pedestrian crashes and fatalities within the preceding five years [6]. Nevertheless, pedestrian-vehicle collisions in Korea remain a serious issue in terms of maintaining pedestrian safety and, ultimately, a high quality of life.

According to statistical data on traffic accidents collected by the Korea Road Traffic Authority, of the 4185 deaths in traffic accidents in Korea in 2017, pedestrian fatalities, at 1675 deaths, accounted for around 40% [7]. The particular severity of pedestrian-vehicle collisions makes pedestrians especially vulnerable as road users [8,9,10].

Moreover, a report by the Korea Centers for Disease Control and Prevention indicates that the average hospitalization period for an injury sustained in a pedestrian-vehicle crash is approximately 4–6 days longer than the average hospitalization period for someone injured in traffic accidents generally [11].

Elderly and child pedestrian-vehicle accidents are the most serious [12]: 54.1% of pedestrian fatalities in 2016 were elderly people over 64 years old [7]. In addition, the number of pedestrian deaths of children under the age of 14 in Korea is second only to that in the United States among OECD countries, indicating the serious danger of poor traffic safety [5].

Previous studies have found that pedestrian age has explanatory power in relation to the severity of injury [1,9,10,13,14]. This is because the physical characteristics and behaviors of pedestrians in urban areas, along roads, vary depending on their age. Consequently, this makes it necessary to examine the association between the severity of the injury and the built environment in relation to pedestrian age, to provide more efficient and effective policy recommendations [15].

This study compares and analyzes models of severity for pedestrian-vehicle crashes by pedestrian age. Furthermore, the study develops differentiations of the characteristics of pedestrian crashes and of the built environment that affect injury severity by various age groups.

## 2. Literature Review

Research on the severity of pedestrian-vehicle crashes has been ongoing for many years in multiple countries [1,2,3,8,9,10,13,14,16,17,18,19,20,21,22,23]. Many studies have identified factors that affect the severity of pedestrian injuries, including the personal characteristics of pedestrians and drivers, temporal characteristics, and aspects of the built environment, such as road characteristics and land use.

### 2.1. Accident Characteristics

The characteristics of pedestrian-vehicle crashes include the personal characteristics of both pedestrians and drivers, and the temporal characteristics of the event. These factors may be taken to characterize the nature of pedestrian crashes in the study area and serve as control variables. In addition, by assessing these factors, it will be possible to grasp the influence of personal characteristics on the severity levels of pedestrian crashes.

The personal characteristics of pedestrians include age and gender. Considering these factors is important because injury severity can vary depending on the physical conditions of a given pedestrian, and can even vary between two individuals injured in collisions under similar environmental conditions. Pedestrian age is often divided into age groups [3,8,9] or classified as children, adults, and the elderly [10,13,22].

Numerous studies show that injury severity varies according to pedestrian age [3,8,9,10,13,14,18,22,24,25,26]. In particular, elderly pedestrians are more likely to be seriously injured compared to those belonging to other age groups. Young pedestrians are more likely to be in good physical condition, are generally quicker to respond, and tend to wear fairly bright clothes [22]; older pedestrians suffer in each of these comparisons and are consequently more likely to suffer fatalities or serious injuries in crashes.

Female pedestrians are more likely to suffer serious injuries compared to men [9,17,22,24,26]. However, Clifton et al. [13] and Abdul-Aziz et al. [14] found inconsistent results in this regard. Other studies have investigated pedestrian behavior at the time of the crash. Kim et al. [1] and Abdul-Aziz et al. [14] state that pedestrians are more likely to suffer serious injuries while crossing crosswalks.

Driver characteristics are considered in a manner similar to that for pedestrian characteristics. Lee and Lee [19] find that elderly drivers are associated with a higher risk of severe injury because they tend to lose physical ability and have lower confidence levels [27]. Conversely, Pour-Rouholamin and Zhou [10], Guo et al. [22], and Choe et al. [28] find that young drivers, who are relatively inexperienced in driving, have a higher risk of fatality and can cause serious accidents. In addition, studies focusing on drivers’ gender find statistically significant differences between male drivers and female drivers in relation to injury severity [1,18,19,21,23].

Drivers’ violations of traffic regulations are closely related to the severity of pedestrian injuries. In particular, drivers’ drinking or drug use [1,3,10,13,18,23,27] and speeding [1,23] are found to cause fatal injuries to pedestrians. Moudon et al. [8], Zahabi et al. [16], and Prato et al. [21] found that driver behaviors in the event of pedestrian-vehicle crashes are related to injury severity.

Additionally, the particular vehicles that are directly involved in pedestrian crashes play a significant role in pedestrian casualty risk. Many studies have included the type of vehicle in their analysis and have found that pedestrian injuries involving a large vehicle, such as a bus, truck, van, or SUV, are more likely to be severe than injuries from crashes involving a passenger car [9,10,18,21,25,26].

The conditions of time and weather at the time of accident are also related to severe injuries among pedestrians. Injury severity tends to be high when pedestrian-vehicle crashes occur at night rather than during the day [3,26]; the same is true for the morning and evening rush hours vis-à-vis other times [9], and weekends vis-à-vis weekdays [3]. Seasonal and weather conditions are also related to the severity of pedestrian-vehicle accidents [2,21].

### 2.2. Characteristics of the Built Environment

Many studies have found that the built environment near the crash, including the land use, facilities, and physical characteristics, have an influence on the frequency and severity of pedestrian injuries.

The characteristics of the roads on which pedestrian-vehicle crashes occur have a significant impact on the severity of injuries. Severe injury to the pedestrian is associated with the conditions of the road [2,10,19], the type of road [10], its hierarchy [2,3,9], the number of lanes [9,10,14,18,20], the posted speed limit [17,21], the location of the accident [8,21,28], and general light conditions [1,2]. Of these, it is found that pedestrian crashes that occur on roads with more lanes [10,14,18,20], on freeways [1,9,23], and at crosswalks and intersections are, in general, the cause of greater damage to pedestrians [9,19].

In addition, the presence of traffic signals in relation to traffic control devices is considered a major variable in several studies [2,10,14,22,24]. Hleem et al. [29] found a difference in pedestrian casualty risk depending on whether there is a traffic signal at the intersection.

Numerous studies have investigated the relationship between public transportation facilities and pedestrian injury severity, and found a significant relationship between the presence of bus stops and the severity of pedestrian injury [13,16,18,19,22]. The presence of a safety zone to protect pedestrians was found to lower the severity of injuries in a study by Seo and Lee [20], but Lee and Lee [19] did not find a significant relationship.

Land use in relation to residential areas, commercial areas, business areas, and industrial areas has been considered, and it is found that the severity of injuries is generally lower in commercial areas [2,14,16,19]. In addition, the areas surrounding parks [16,18], parking facilities [14,21], and hospitals [21] are associated with greater injury severity.

Several studies have been conducted to identify the socioeconomic factors affecting the severity of pedestrian injuries. Income [18,22] and race [21,30,31] are related to the severity of pedestrian-vehicle crashes. As mentioned above, pedestrian age is a major factor in the degree of injury severity. Demetriades et al. [32] indicated that the degree of severity differs by pedestrian age. In addition, a recent study by Toran Pour et al. [15] showed that the temporal and spatial distributions of pedestrian-involved accidents vary according to the age of the pedestrian.

However, although some studies [19,33,34] have focused on the age of the driver, few studies have focused on the age of the pedestrian. Unlike previous works, which largely focus on drivers, this study investigates the association between the built environment and pedestrian-involved accidents by drawing attention to vulnerable road users (i.e., children and the elderly) to enable the construction of a safe walking environment.

### 2.3. Methods of Injury Severity Studies

Over the past years, researchers have developed various research methods to assess the severity of pedestrian-vehicle crashes. Injury severity is typically classified on an ordinal scale according to the degree of pedestrian injury. For example, a number of studies have used the KABCO (K = fatal injury, A = incapacitating injury, B = non-incapacitating evident injury, C = possible injury, and O = no injury) injury recording system [8,25,27]. Furthermore, several studies on the severity of pedestrian crashes have classified injury severity into two classes and analyzed logit-form models. Different models have been obtained and applied, such as the binary logistic model [3,24,26,28,35,36,37,38,39,40], multinomial logit model [9,41], mixed logit model [23,34,42,43], ordered logit/probit model [10,13,18,19], and ramdon parameters bivariate ordered probit model [44]. The binary logistic model is one of the most common approaches used in studies on injury severity. Specifically, Cai et al. [35] developed a binary logistic regression model for the analysis of pedestrian and bicycle crashes at the micro-level. Sun et al. [37] investigated the factors that influence the injury severity from traffic accidents in Beijing using the binary logistic regression model. Other studies on injury severity have utilized the mixed logit model to capture unobserved heterogeneity in crash data [43]. Chen and Chen [42] employed a mixed logit model to identify and explore the injury severity of truck drivers involved in single- and multi-vehicle accidents on rural highways [42]. Pour-Rouholamin et al. [10] and Mohamed et al. [18] investigated the risk factors associated with pedestrian injury severity in Illinois and New York. Recently, Chen et al. [44] utilized a random parameter bivariate ordered probit model to analyze the severity of injury to drivers involved in rear-end collisions between cars.

In terms of research methodology, various methods using new technology have been evaluated in recent years to understand the behavior of drivers or pedestrians and road conditions. Hanson et al. [17] utilized a visual inspection of Google Street View imageries to elucidate the manner in which the features of road infrastructure influence pedestrian casualties. Micucci and Sangermano [45] employed video cameras to examine whether road users follow traffic laws and argued that the behavioral characteristics of cyclists have a significant influence on road safety. Micucci et al. [46] used recording videos shot by a helmet’s 360-degree camera and a virtual reality visor to survey the association between turning signal detection and motorcyclist characteristics.

## 3. Materials and Methods

### 3.1. Study Area and Analytical Method

**Figure 1:**
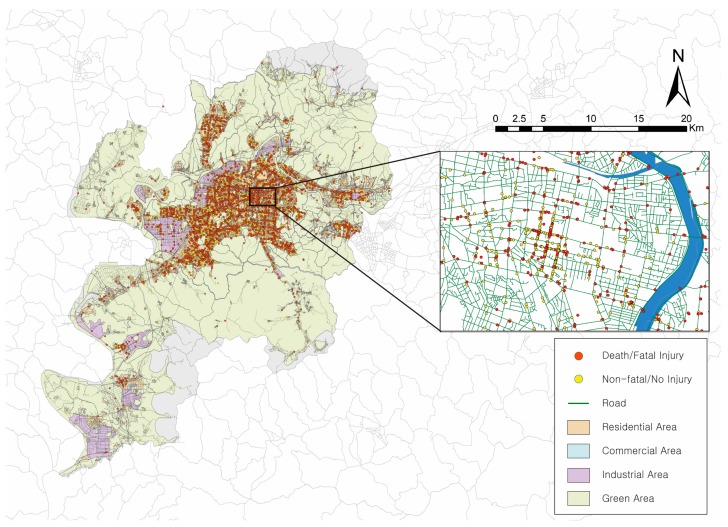
Daegu Metropolitan City and the spatial distribution of pedestrian-vehicle crashes.

**Figure 2:**
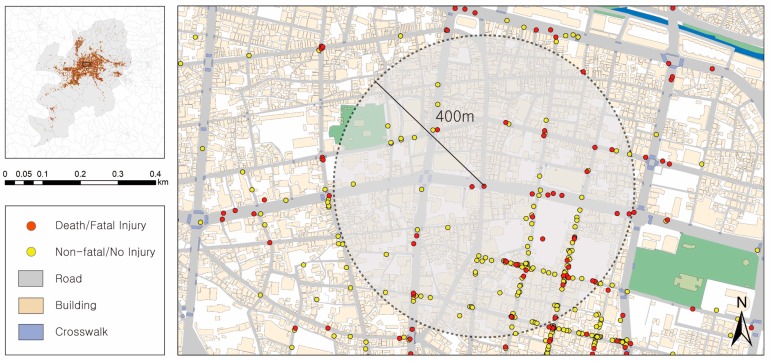
An example of the 400 m radius around a pedestrian crash.

This study investigates factors that affect the severity of pedestrian injuries in pedestrian–vehicle crashes in Daegu Metropolitan City, which is the fourth most populous city in Korea. Daegu’s total area is 883.54 km^2^, and its population is around 2.47 million. The spatial distribution of pedestrian-vehicle crashes is shown in Figure 1. The spatial unit of analysis is a 400 m radius centered on the point of the crash (Figure 2). Table 1 shows the structure and definitions of the variables considered in the analysis. Table 1 provides the definitions and structures of the variables considered in the analysis and presents the descriptive statistics for the 7881 samples.

In the raw data of pedestrian-vehicle crashes, the severity of pedestrian injuries is classified into four categories, namely, death, fatal injury, non-fatal injury, and possible injury. The study aims to identify the determinants of pedestrian fatality increases. Thus, the dependent variable is defined as individuals falling under one of two groups, namely, non-fatal injury or possible injury (=0) and death or fatal injury (=1).

Binary logistic regression analysis was used to consider the dummy dependent variable. This type of analysis, in which a dependent variable takes a value of 0 or 1, is a widely used approach in research on the severity of traffic accidents [24,26,28,35,36,37,38,39,40]. This approach differs from general regression analysis because it proves the probability of the incidence of pedestrian fatalities corresponding to the assigned value of the built environment observed.

The binary logistic regression model is presented as follows [40,46,47]:

(1) $$Odds=\frac{p}{1-p}$$

(2) $$\ln\left(\frac{p}{1-p}\right)=\alpha +\beta_{1}X_{1}+\beta_{2}X_{2}+\cdots +\beta_{n}X_{n},$$

where the odds ratio indicates the probability of the occurrence of a severe pedestrian-vehicle crash p. In binary logistic regression, p refers to the severity of pedestrian injury. α denotes a constant, whereas β is the independent variable.

### 3.2. Accident Data

The dataset for pedestrian crashes examined in the study was derived from the Traffic Accident Analysis System (TAAS) of the Korea Road Traffic Authority and was geocoded using the Geographic Information System (GIS). The dataset of traffic accidents used in this study was taken from Korea Road Traffic Authority. These data include accident characteristics of both pedestrians and drivers, the severity level of the injuries, vehicle information, and the temporal and locational factors at the time of the event. From this dataset, pedestrian-vehicle crashes were extracted. The study categorized pedestrians into five age groups, namely, under 20 years old (children/adolescents), 20–39 years old (young), 40–64 years old (middle), over 64 years old (old), and the complete group with all age groups combined. The classification was based on vehicle driving availability and travel mode choice, thus reflecting the social and cultural characteristics of South Korea.

The gender of the pedestrians and drivers was constructed with a dummy variable. Ages were entered as a continuous variable. The vehicles involved in pedestrian-vehicle collisions were defined by a dummy variable in the following manner: 0 = a passenger car, and 1 = a van or truck. To identify the influence of time and weather; this study incorporated the variables of day versus night; and clear versus cloudy, rainy, snowy, or foggy weather as dichotomous dummy variables.

### 3.3. Neighborhood Data

The characteristics of the built environments in neighborhoods consist of safety zone, road, and land use characteristics. The safety zones are divided into school zones, and silver zones for the safe walking of vulnerable road users, such as children and the elderly. The school zones and silver zones, which are emblematic of pedestrian safety policies in Korea, are legally designated safety zones that were established to protect children and seniors from traffic accidents under the Road Traffic Act [48]. In Korea, school zones are designated within a 300-meter radius of an elementary school or a preschool, whereas silver zones are situated within a radius of 300 m from an elderly welfare facility [48]. Both variables were accounted for because the 400 m area around the spot of the pedestrian crash was considered in each case (Figure 2).

The number of bus stops is used to determine the link between public transit and pedestrian crashes. Crosswalks and traffic signals that could be expected to affect the behavior of both pedestrians and drivers were included in the analysis. Speed-control cameras and posted speed limits, both of which may keep drivers from speeding, were also studied in this research. Road types, neighborhood streets, and arterial roads were also considered in order to determine any connection to pedestrian-vehicle crashes. Finally, immediately adjacent land use, whether residential, commercial, or industrial, was examined.

The dataset pertaining to the neighborhood environment was provided by various governmental agencies. The variables for crosswalks and land use were obtained from the National Spatial Data Instructure Portal. The posted speed limit can be accessed on the website of the Intelligent Transportation System of the National Transport Information System. Road attributes can be accessed from the Road Name Address Information System website. Other data, which are not open to the public, were obtained through direct contact with the Department of Transportation of the Daegu Regional Government. All datasets pertaining to neighborhood environment were also geocoded using GIS.

## 4. Results and Discussion

Table 2 and Table 3 show the results of a logistic regression analysis of the severity of pedestrian-vehicle crashes in Daegu from 2013 to 2015. Model 1 gives the analysis of all pedestrian crashes regardless of pedestrian age. Model 2 gives the results for the age group of under-20-year-olds, Model 3 covers the age group of 20–39-year-olds, Model 4 describes the age group of 40–64-year-olds, and Model 5 presents the age group of over-64-year-olds.

### 4.1. Pedestrian Age Groups and the Severity of Pedestrian Injuries

The combined group of all age groups exhibited more statistically significant relationships than the other models did. In particular, the variable of pedestrian age exhibited a significant influence on pedestrian crashes resulting in severe injuries. As has been found in many studies [1,2,3,8,10,17,21,26], severe injuries are more common with older pedestrian ages. In addition, the comparison of the values of −2 log-likelihood, which can be used to assess the significance of the pedestrian age parameter and to compare the explanatory power of each model [47], shows that Models 2–5 have higher explanatory powers than Model 1. This finding matches the intention of the study, that is, to find the occurrence of severe injuries by pedestrian age group.

The remaining specific age groups indicate that the injury severity of pedestrian crashes shows a different aspect in relation to the age group of the pedestrians. The youngest group shows fewer statistically significant variables than the other models. This shows the distinctiveness of traffic crashes involving children and young pedestrians. This result may relate to some other factors that are not considered in this model. It also implies that there may be limitations to the explanation of severe injury in accidents with children and youth pedestrians with respect to the characteristics of pedestrians, drivers, and the built environment of the neighborhood.

Conversely, the severity of traffic accidents involving the remaining groups of pedestrians aged 20 years or older is found to be influenced by various accident characteristics and factors of the built environment. In particular, the characteristics of the built environment that affect injury severity are different for each age group of pedestrians. This result suggests that traffic crashes with adult pedestrians could be prevented and reduced through adequate maintenance of the built environment. This result also implies that differentiated policies and strategies are needed to target the prevention of pedestrian-vehicle crashes in relation to the age of pedestrians.

With the oldest group, personal and time factors have more explanatory power than the built environment. Elderly pedestrians have a lower level of physical abilities and are less capable of coping with dangerous situations than other age groups [2]. Demetriades et al. [32] argued that pedestrian age has a significant effect on the ability to recover and achieve survival outcomes after an accident, and in traffic accidents, the elderly are more likely to suffer serious injuries in vital areas such as the head, thorax, and spine than other age groups. This suggests that more detailed and intensive research is required to reduce the probability of traffic accidents, and to reduce mortality in elderly pedestrian-vehicle crashes in an aging society.

### 4.2. Accident Characteristics

The statistically significant factors for severe injury are different in each age group. Pedestrians’ gender is not significant in the age group of under-20-year-olds, but in the other groups, women are more likely to be seriously injured from pedestrian-vehicle collisions. Previous studies have found a higher risk of serious injury in female pedestrian-involved accidents [9,13,24].

Driver gender has a significant relationship with the severity of injury in both groups over 40 years old, and male drivers produce more severity. This result generally confirms the findings in the literature [1,3,19,21].

Regardless of pedestrian age, the higher the driver’s age, the higher the severity of injury among pedestrians. This finding is in agreement with previous studies [19]. Vehicle type is not significantly correlated with the likelihood of severity of injuries in any age group, which is not consistent with the literature [3,9,10,21,23,25,26]. This inconsistent result may imply that personal characteristics, such as the age and gender of the driver, play a more important role in accident severity.

Other characteristics of the driver’s driving behavior, such as driving experience [3,28], drunk driving [9,13,17,18,23,27], and speeding [1,3,23], which have been considered in previous studies, were not considered in this study because of the limitations of data acquisition in Korea. Nevertheless, because the personal characteristics of pedestrians and drivers cannot be directly controlled, a more direct strategy might be to build a safe walking environment through policy development that is oriented toward the built environment.

Furthermore, adult pedestrians are more likely to sustain serious injury at night than they are during the day. As in many previous studies [2,3,13,18,21], this may be because both drivers and pedestrians experience poor vision at night. Likewise, drunk driving and speeding occur more often at night. However, the difference between day and night is insignificant in the youngest group.

The impact of weather on injury severity is strongly significant only for the age group of 20–39-year-olds. Pedestrian injury is more likely to be severe in cloudy, snowy, or rainy weather than it is in clear weather, which is similar to the corresponding findings of previous studies [9,29]. Tay et al. [9] also conclude that both pedestrians and drivers have poor sight in poor weather conditions, as well as at night.

However, Yu [2] and Jang [49] show a greater risk of pedestrian injury in clear weather, concluding that in clear weather, drivers tend to be less careful, and pedestrians are less likely to walk in inclement weather [2]. These inconsistent results require further investigation.

### 4.3. Built Environmental Characteristics

In general, the characteristics of the physical environments of neighborhoods show more distinctive differences by age group than do the individual characteristics involved in pedestrian crashes. Among all pedestrian-vehicle crashes, various characteristics of the built environment in neighborhoods affect pedestrian injury severity. However, within the age groups, the influence of the characteristics of the built environment in the neighborhoods on the risk of pedestrian injury is clearly differentiated. These findings show that to prevent pedestrian-vehicle crashes more effectively, concrete and constructive policies must be deployed that take the age of pedestrians into account, rather than general integrated policies.

First, school zones and silver zones to protect the vulnerable elderly and children show dissimilar results by age group. An interesting result is that the school zones, designated for the safety of children, have a positive relationship with the severity of pedestrian injuries. The larger the area of the school zone, the more likely the pedestrian crash is to be severe. The same result is also shown in the age group of 40–64-year-olds. It is concluded that this is due to the parents being injured while picking up their children around schools, which is why there are fewer severe injuries in this area relative to the other age groups. This result raises questions regarding the effectiveness of school zones, a symbolic policy for pedestrian safety in Korea. Clearly, more effective policy development is needed for school zones. Silver zones show no statistical significance in any group except in the age group of 40–64-year-olds. In particular, it is worth noting that silver zones are not significantly correlated with the severity of pedestrian injuries in the oldest group. School zones and silver zones are designed to reduce pedestrian-vehicle collisions and to protect vulnerable road users. Beyond merely designating a protection zone for speed reduction, integrated strategies must be developed that can consider all nearby systems, programs, and policies for protecting young and elderly pedestrians in the protection zone.

Bus stops, used here to indicate public transportation density, which may promote walking, fail to have statistical significance in all models. However, Mohamed et al. [18] and Chen and Zhou [50] indicate that the bus stop is positively related to the severity of pedestrian injuries and the occurrence of pedestrian-vehicle collisions. This unexpected result, which is generally recognized as encouraging walking behavior, requires an in-depth study of the pedestrian environment around bus stops.

For all age groups and for the age group of 20–39-year-olds, severe injuries are less common where the number of crosswalks around the accident spot is higher. Places with many crosswalks are generally intersections or pedestrian-priority roads where vehicles drive relatively slowly, and where drivers tend to pay closer attention to their driving. However, Seo and Lee [20], who investigated Seoul, a similar research location, found conflicting results. Therefore, a more detailed and in-depth investigation is needed that includes a comparison of crosswalks that have traffic signals with crosswalks that do not.

The number of traffic signals shows statistical significance only for the oldest pedestrians. However, the more common traffic signals are around the spot of the accident, the greater the severity of pedestrian injuries. Areas with more traffic signals are likely to be areas with many intersections, crosswalks, and neighborhood streets rather than arterial roads. In these areas, the pedestrian volume is likely to be high, and vehicle speeds tend to be slow. A significant association is found only in the elderly group and can be judged to be increased by the variant physical characteristics of the elderly within the same environmental conditions that others face. Elderly pedestrians tend to ignore signals at crosswalks with traffic signals and do not keep pace with changing traffic signals because of physical discomfort. Specifically, 54.1% of the victims of South Korean pedestrian fatalities are elderly pedestrians, and this group tends to walk against signals [7]. Bae and Park [51] indicate that crosswalks with traffic signals can cause jaywalking. Therefore, it is recommended that pedestrian safety strategies be developed that take into account the characteristics of the elderly and their physical conditions. In particular, policy development and interventions in connection with the silver zone discussed above are required.

The presence of speed cameras is negatively related to pedestrian injury severity in the set of all age groups and in the age group of 20–39-year-olds. Yoon et al. [52] demonstrate that speed cameras contribute, in part, toward reducing the severity of pedestrian-vehicle crashes. However, they fail to reduce the likelihood of severe injury in pedestrians among the young and elderly. Therefore, the positive effects of speed cameras must be linked to the school and silver zones to improve the walking safety of vulnerable road users.

In a similar vein, the higher the posted speed limit of vehicles on the road in the set of all groups is, the higher the chance of severe pedestrian injuries. This result is in agreement with previous studies, suggesting the need for the strong and continuous management of speeding vehicles [2,17,20,21,23,24]. However, it is interesting that an assessment by age group does not show a statistically significant difference according to the average posted speed limit.

The severity of pedestrian injury is high in areas with more neighborhood streets in the set of all groups and the age group of 40–64-year-olds. However, arterial roads do not show statistically significant results for any model. This is presumably because there are more pedestrians on neighborhood streets, which are primarily designed for them, than there are on the arterial roads, which are built for vehicle traffic, and where the frequency of exposure to pedestrian-vehicle collisions is reasonably high. This contradictory result follows previous studies [13,50]. More actual micro-level research that involves field surveys, such as accounting for roadside trees, sidewalks, pavements, and roadside facilities, is required to understand how to build a safe walking environment on neighborhood streets, where pedestrian traffic, not vehicle traffic, is the main focus.

Land use has results that do not support previous studies [50,53,54]. Here, residential and commercial areas are not associated with the likelihood of severe pedestrian injuries. Conversely, the greater the industrial areas in the set of all groups and the age group of 40–64-year-olds are, the higher the severity of pedestrian injuries is. Industrial land use features large and heavy vehicles more often. Although this study fails to show any significant correlation with this, many studies that investigate vehicle characteristics find that heavy vehicles tend to increase the severity of pedestrian injuries [21,23,25]. In particular, the age group of 40–64-year-olds, which showed a significant correlation for severity in crashes, unlike other age groups, appears to illustrate the fact that this is also the age group of many workers in Daegu’s industrial areas. Lee and Lee [19], who studied Seoul, provided consistent results, but Chen and Zhou [50], who studied Seattle, revealed contrary findings. Therefore, it is necessary to understand the impact of the severity of pedestrian-vehicle crashes depending on the country or area, and to develop differentiated policies and strategies that consider the distinguishing characteristics of the country or city to build a safe pedestrian environment.

## 5. Conclusion

This study examines the factors that affect pedestrian-vehicle crashes with the intention of ultimately creating an environment where pedestrians can walk safely. In particular, to reduce the severity of pedestrian injuries when pedestrian accidents occur, this study investigates the crash characteristics and factors of the physical environment that affect the likelihood of the severity of pedestrian–vehicle crashes.

In addition, the significance of this study lies in the observation that the results are derived through a consideration of pedestrian behaviors in relation to age group, which has been neglected in previous research. The main results and implications of the study can be summarized as follows.

First, factors affecting the likelihood of severe injury in a pedestrian-vehicle crash show variant patterns by age group. Various features in the set of all groups have statistical significance for the severity of pedestrian injuries, but their statistical significances are meaningfully different in different age groups. Among the age group models, the age group of 40–64-year-olds has the most significant correlations with the aspects we investigated, whereas the young age group shows the fewest statistically significant variables. These results indicate that age-based policy development and strategies are needed for more efficient and powerful policies, particularly for vulnerable road users, such as children and the elderly, rather than analyses that are based on general aggregated crash data.

Second, the personal characteristics of the drivers and pedestrians and the time characteristics of the occasions of pedestrian-vehicle crashes are crucial factors that affect the likelihood of severe pedestrian injuries, with the exception being the age group of under-20-year-olds. Injury severity is high when the driver is male and when the pedestrian is female. Therefore, it is necessary to investigate the characteristics of behaviors according to the genders of the drivers and the pedestrians to establish policies for pedestrian safety. Moreover, the driver’s age is a significant crash indicator in all models. The older the driver is, the more likely a severe pedestrian injury is. This is because, as with pedestrian age, the driver’s physical condition and ability to cope with pedestrian crashes decreases with age. Pedestrian crashes that occur at night, rather than during the day, are highly correlated with highly severe injuries. Therefore, more direct and diversified policy approaches are required, such as expanding safety facilities and securing the visibility of road facilities for safer night driving and safer driving among older drivers.

Third, the factors of the physical environment related to the severity of pedestrian injuries exhibit distinct differences by age group, which can be understood as being related to particular walking behaviors, road use, and the physical characteristics of urban spaces inhabited by different age groups. Therefore, a study on differences in walking and driving behaviors by age group might indicate a need for convergence research in various fields, such as public health, urban planning, and urban design.

Finally, the influence of school zones and silver zones, which are representative policies adopted in Korea for pedestrian safety, differs by age group and, on average, tends to increase the likelihood of severe pedestrian injury or is not statistically significant. Therefore, the effectiveness of these policies must be closely reviewed through future research, and practical policies and interventions are needed.

In this study, empirical research is conducted specifically on the differences in the likelihood of severe injury by age group. However, because pedestrian-vehicle collisions take place in space, there is a limitation in that this study does not consider spatial autocorrelation even though data with spatial attributes are used. The severity of pedestrian injury can be influenced by driver behavior (e.g., drunk driving) and pedestrian behavior (e.g., jaywalking). However, the study did not fully consider driver and pedestrian behaviors because of the difficulty in acquiring the data, which can be considered to be a limitation of the study. In addition, the binary logistic model, which has been widely used in recent studies, was used to analyze data in which the dependent variable was in logit form. However, another limitation of the study is that unobserved heterogeneity in the crash data was not taken into account. Future research should address these limitations, which will require additional accurate and specific parameters.

Walking is the most basic means of transportation for human beings; it is also the healthiest and most sustainable one. Creating a safe walking environment is a vital aspect of enabling a high quality of life. In addition to the present investigation, further research is needed, and more in-depth policies for pedestrians and drivers to share roads safely and conveniently should be discussed.

## Figures and Tables

**Table 1:** Construction of the variables used in the analysis and basic statistics.

Variables	Measurement	Mean (SD)
**Dependent Variables**		
Severe injury, all pedestrians	1 = death, fatal injury; 0 = non-fatal injury, possible injury	0.47 (0.50)
Severe injury, 0–19-year-old pedestrians		0.34 (0.47)
Severe injury, 20–39-year-old pedestrians		0.32 (0.47)
Severe injury, 40–64-year-old pedestrians		0.48 (0.50)
Severe injury, over-64-year-old pedestrians		0.67 (0.47)
**Independent Variables**		
Individual Characteristics		
Pedestrian Variables		
Pedestrian age	Continuous (year)	44.07 (22.16)
Pedestrian gender	1 = male; 0 = female	0.54 (0.50)
Driver Variables		
Driver age	Continuous (year)	44.95 (15.47)
Driver gender	1 = male; 0 = female	0.73 (0.45)
Vehicle type	1 = van, truck; 0 = passenger car	0.20 (0.40)
Temporal Variables		
Time	1 = night time (6pm–6am); 0 = day time (6am–6pm)	0.50 (0.50)
Weather condition	1 = cloudy, rain, snow, fog, other; 0 = clear	0.13 (0.33)
Neighborhood Characteristics		
Safety Zones		
School zone	Total area of school zones in 0.4 km radius	259,455 (177,535)
Silver zone	Total area of silver zones in 0.4 km radius	40,951 (78,112)
Road Environments		
Bus stop	Number of bus stops in 0.4 km radius	7.75 (3.24)
Crosswalk	Number of crosswalks in 0.4 km radius	37.43 (18.41)
Traffic signal	Number of traffic signals in 0.4 km radius	32.10 (14.57)
Speed camera	Number of speed cameras in 0.4 km radius	2.46 (3.61)
Average posted speed limit	Average of posted speed limits in 0.4 km radius	54.89 (7.74)
Neighborhood street	Total length of neighborhood streets in 0.4 km radius	101,412 (47,080)
Arterial road	Total length of arterial roads in 0.4 km radius	10,319 (7358)
Land Uses		
Residential area	Total area of residential use in 0.4 km radius	315,527 (137,196)
Commercial area	Total area of commercial use in 0.4 km radius	97,362 (124,992)
Industrial area	Total area of industrial use in 0.4 km radius	15,929 (65,482)

**Table 2:** The results of binary logistic regression.

Variables	Model 1 (All age groups)			Model 2 (Under-20-year-old group)		
	*β*	Odds Ratio	*p*-Value	*β*	Odds Ratio	*p*-Value
**Individual Characteristics**						
Pedestrian Variables						
Pedestrian age	0.024 ***	1.024	<0.001			
Pedestrian gender	−0.391 ***	0.677	<0.001	−0.031	0.969	0.782
Driver Variables						
Driver age	0.009 ***	1.009	< 0.001	0.009 ***	1.009	0.009
Driver gender	0.162 ***	1.176	0.005	0.055	1.056	0.670
Vehicle type	0.004	1.004	0.951	−0.069	0.933	0.655
Time	0.265 ***	1.304	<0.001	0.080	1.084	0.493
Weather condition	0.194 ***	1.214	0.007	0.108	1.115	0.570
**Neighborhood Characteristics**						
Safety Zones						
School zone	0.000 ***	1.000	0.005	0.000 **	1.000	0.030
Silver zone	0.000	1.000	0.282	0.000	1.000	0.922
Road Environments						
Bus stop	−0.013	0.988	0.187	−0.032	0.969	0.134
Crosswalk	−0.003 *	0.997	0.093	−0.005	0.995	0.277
Traffic signal	0.003	1.003	0.158	−0.001	0.999	0.856
Speed camera	−0.013 *	0.987	0.083	0.014	1.014	0.470
Average posted speed limit	0.006 *	1.006	0.061	0.001	1.001	0.916
Neighborhood street	0.000 **	1.000	0.013	0.000	1.000	0.211
Arterial road	0.000	1.000	0.579	0.000	1.000	0.178
Land Uses						
Residential area	0.000	1.000	0.935	0.000	1.000	0.953
Commercial area	0.000	1.000	0.311	0.000	1.000	0.599
Industrial area	0.000 **	1.000	0.019	0.000	1.000	0.940
Constant	−1.530 ***	0.217	<0.001	−0.401	0.670	0.453
number of observations	7881			1498		
−2 log likelihood	10,067.93			1880.20		

* *p* < 0.1, ** *p* < 0.05, *** *p* < 0.01.

**Table 3:** The results of binary logistic regression.

	Model 3 (20–39-year-old group)			Model 4 (40–64-year-old age group)			Model 5 (Over-64-year-old age group)		
	*β*	Odds Ratio	*p*-Value	*β*	Odds Ratio	*p*-Value	*β*	Odds Ratio	*p*-Value
**Individual Characteristics**									
Pedestrian Variables									
Pedestrian gender	−0.394 ***	0.674	<0.001	−0.530 ***	0.589	<0.001	−0.449 ***	0.638	<0.001
Driver Variables									
Driver age	0.010 ***	1.010	0.007	0.007 **	1.007	0.013	0.009 ***	1.009	0.004
Driver gender	0.015	1.015	0.910	0.267 ***	1.306	0.009	0.297 ***	1.346	0.008
Vehicle type	0.006	1.006	0.969	0.101	1.107	0.355	−0.082	0.921	0.454
Temporal Variables									
Time	0.616 ***	1.851	<0.001	0.506 ***	1.658	<0.001	0.218 **	1.244	0.031
Weather condition	0.405 ***	1.499	0.005	0.132	1.141	0.264	0.270 *	1.311	0.083
**Neighborhood Characteristics**									
Safety Zones									
School zone	0.000	1.000	0.724	0.000 **	1.000	0.044	0.000	1.000	0.158
Silver zone	0.000	1.000	0.889	0.000 **	1.000	0.046	0.000	1.000	0.692
Road Environments									
Bus stop	−0.022	0.979	0.322	−0.001	0.999	0.939	−0.014	0.986	0.450
Crosswalk	−0.008 *	0.992	0.059	0.000	1.000	0.999	0.003	1.003	0.538
Traffic signal	0.008	1.008	0.137	−0.006	0.994	0.138	0.008 *	1.008	0.087
Speed camera	−0.040 **	0.961	0.016	−0.005	0.995	0.683	−0.012	.988	0.405
Average posted speed limit	0.010	1.010	0.172	0.007	1.007	0.221	0.003	1.003	0.665
Neighborhood street	0.000	1.000	0.325	0.000 ***	1.000	0.005	0.000	1.000	0.575
Arterial road	0.000	1.000	0.230	0.000	1.000	0.639	0.000	1.000	0.981
Land Uses									
Residential area	0.000	1.000	0.491	0.000	1.000	0.286	0.000	1.000	0.304
Commercial area	0.000	1.000	0.943	0.000	1.000	0.350	0.000	1.000	0.101
Industrial area	0.000	1.000	0.362	0.000 **	1.000	0.017	0.000	1.000	0.220
Constant	−1.353 ***	0.258	0.005	−0.533	0.587	0.175	0.166	1.181	0.685
number of observations	1731			2452			2200		
−2 log likelihood	2063.61			3278.50			2727.86		

* *p* < 0.1, ** *p* < 0.05, *** *p* < 0.01.
